# Simulation as a potential tool for successful clinical trial initiation

**DOI:** 10.1017/cts.2024.559

**Published:** 2024-05-31

**Authors:** Rita Dadiz, Rachel Jones, Ronnie Guillet

**Affiliations:** Division of Neonatology, Department of Pediatrics, Golisano Children’s Hospital, University of Rochester Medical Center, Rochester, NY, USA

**Keywords:** Clinical trial initiation, clinical trials, communication, neonatal research, simulation

## Abstract

Conducting clinical trials is often complex and involves many individuals from a variety of services, each with a specific role in ensuring its successful implementation. Although an experienced clinical trialist may anticipate many of the challenges, others may be unexpected and detrimental to the successful completion of a study. We describe the use of simulation during preparation for initiation of a randomized clinical trial of a new preparation of antiseizure medication in neonates with seizures. The process of identification of stakeholders and roles, scenario development, and identification of challenges are described. Lessons learned included the potential benefits of simulation exercises, simulation challenges, and challenges associated with the study itself. We posit that going through the steps of a study, rather than merely reading them from a manual of procedures, will help identify potential barriers, complexities, and contingencies that are not readily apparent and may result in fewer protocol deviations and violations.

## Introduction

Multicenter clinical trials are essential for advancing both our understanding of pathophysiology and how best to evaluate and treat patients. Conducting clinical trials is often complex and involves many individuals from a variety of services who each have a specific role in ensuring its successful implementation throughout the study period. Identification of appropriate subjects (“screening”), approach for consent, and enrollment may be time limited within a very short window. Coordination of all scheduled interventions and procedures may also be a challenge due to the need for specialized personnel interacting with the subject as well as with each other in a very defined sequence. Although an experienced clinical trialist may anticipate many of these challenges, others may be unexpected and detrimental to the successful completion of a study.

Simulation may be applied as a tool to assist study teams in anticipating potential challenges, developing and rehearsing strategies to enhance communication among team members, and solidifying workflow processes before study initiation. In healthcare, simulation has been utilized in education to improve knowledge, technical skills, and clinical decision making, especially in the rehearsal of high acuity, low occurrence clinical situations [1–5]. Simulation has also been utilized in quality improvement and assurance to effectively identify and mitigate latent safety threats in novel situations and workplace environments, allowing healthcare teams to design clinical spaces, communication practices, and workflow processes to deliver safe patient care [6–11]. To our knowledge, simulation has not been employed as an initial step to prepare study teams for beginning a complex clinical trial. We had the opportunity to conduct simulations to prepare for a new randomized clinical trial, during which we aimed to identify potential study-related issues during the screening, enrollment, and initial study procedures. In this article, we present our process, as well as describe the challenges faced and lessons learned.

## Methods

### Initial steps

In 2022, researchers at the University of Rochester were invited to join a randomized clinical trial to evaluate the pharmacokinetics of a new preparation of a commonly used antiseizure medication and investigate the efficacy of two different dosages in infants. The study was approved by faculty in the Division of Neonatology and the Institutional Review Board. Neonatal providers were assured that all subjects would receive treatment, and the difference between treatment arms was the dosage of the medication.

During discussions regarding study logistics among the investigators and the study coordinator, it became clear that the timeline for identifying, consenting, and enrolling study participants, as well as for initiating the study drug, was very short and involved the close and effective coordination of several different stakeholder groups. Given the subject population and the clinical diagnosis, the individuals needed to successfully carry out all study procedures would have to be readily available at any time. Thus, a small team consisting of a neonatologist with expertise in neonatal neurology and conducting clinical trials, the research coordinator, and a neonatologist with expertise in utilizing simulation for education, quality improvement, and research carefully reviewed the study protocol, familiarized themselves with all aspects of the trial, and planned simulations to identify barriers to success.

### Identification of stakeholders and roles

For this particular study, different stakeholder groups with specific roles would need to coordinate with each other to help ensure a smooth workflow within the constraints of a tight timeline to identify and enroll potential high-risk infants before they exhibit electrographic seizure activity. In addition to the core research team (site principal investigator, co-investigators, and site coordinator) who would be overseeing the study and the neonatal intensive care unit (NICU) clinical team (physicians, nurses, and other staff) who would be providing routine care to the infant, we identified several services that would have key roles, including child neurology, electroencephalography (EEG) (technicians and certified EEG interpreters), and the investigational drug service (IDS) with the help of pediatric pharmacy.

As part of study procedures, the clinical team identifies neonates likely eligible for the trial and contacts the core research team. The study coordinator or site investigator then reviews the medical record to assess eligibility and obtain permission from the infant’s clinical team to approach the family to introduce the study. During the time the family considers whether or not to participate, the research team notifies the child neurology and EEG teams that there is a potential study participant who, if the parents consented, would need a qualifying EEG to be performed and interpreted. After consent, while preparations for the EEG are underway, the research team contacts IDS to begin preparing the two dosages of the study drug in the event the EEG interpretation indicates that the infant is eligible for study participation. Once eligibility is confirmed by EEG, the research team randomizes the infant to identify which dosage of the study drug the infant would receive. The research team also communicates with the clinical team about pertinent study procedures, including instructions on how to administer the study drug and which laboratory studies to obtain. A qualified examiner performs physical exams and records findings at predetermined intervals. Throughout this process, the research team provides oversight and guidance.

### Development of scenarios

To identify the potential major issues of conducting this study in situations of varying complexity, we designed two simulation scenarios with different infant diagnoses, clinical presentations, timing of seizure activity, and availability of research stakeholders (Supplementary Material 1). We iteratively reviewed and revised the scenarios with input from an additional neonatologist and neurologist to consider conditions that would optimize our ability to uncover challenges.

The goal of the first scenario was to provide participants the opportunity to identify potential issues from subject identification, consent, and enrollment to the initiation of study procedures in a relatively straightforward situation. To allow participants to focus first on basic steps, the scenario occurred during daytime work hours, which eliminated the complexities inherent in off-hour communications. The scenario consisted of a newborn infant who was born on a weekday morning and diagnosed with hypoxic ischemic encephalopathy (HIE). We chose this diagnosis because approximately half of infants with HIE develop clinically apparent seizures at 12–24 hours of age [12,13] and are anticipated to constitute at least half of the study subjects. In this clinical scenario, the occurrence of seizures can be anticipated, and there would be adequate time to speak with parents and communicate with all stakeholders.

The second scenario involved more complexity, building on the lessons learned from debriefing the events of the first simulation. This scenario included a shorter timeline for enrollment and study initiation, as well as an off-hour diagnosis of neonatal seizures to identify issues specific to the performance of study procedures at night or during the weekend. The infant in this scenario unexpectedly presented with seizure-like activity, which was likely due to intracranial hemorrhage or meningitis as the next most common etiologies of seizures in this age group [12,13]. Given the unexpected onset of symptoms and the usual practice of initiating treatment as soon as possible, either following confirmation of electrographic seizures by EEG or more quickly if physiologically destabilizing clinical seizures occurred, the notification of research team members had to be more immediate such that screening, approach, consent, and randomization did not interfere with clinical standard of care.

### Identification of challenges with team-based simulations and debriefings

Individuals representing each of the stakeholder groups were invited to participate in a team-based simulation and debriefing session. Before participation, participants received an email that oriented them to the purpose of the simulation sessions. They also reviewed and had an opportunity to ask clarifying questions about the research protocol.

The simulations were conducted with a moderate-to-high level of fidelity, mirroring as much as possible what would occur in a real clinical situation to achieve the goals and objectives of examining the complex interactions of different stakeholders involved in this study. Because of our focus, it was important that we conducted the simulations *in situ*, in a patient room in the neonatal intensive care unit, with individuals responsible for clinical care and for all study procedures. Participants were instructed to interact with the infant mannequin, utilize computer interfaces, and communicate with each other as they normally would (e.g., face-to-face, text messaging, and web paging). Most parts of the scenario unfolded in real time to gauge the amount of time it would take to accomplish specific tasks. However, for some tasks that would take a longer period of time to complete (e.g., placement of EEG leads and preparation of the different dosages of study drug), participants estimated the length of time based on prior experiences of patient care and/or during clinical trials so that the simulation would proceed more efficiently. Since the simulation was not focused on the procedures themselves but rather on efficient communication among the various services, and performing all the individual steps would require an additional 1–2 hours, we felt this “short cut” was appropriate. For clinical trials that include novel procedures, such procedures can be incorporated into the simulation.

**Figure 1.**
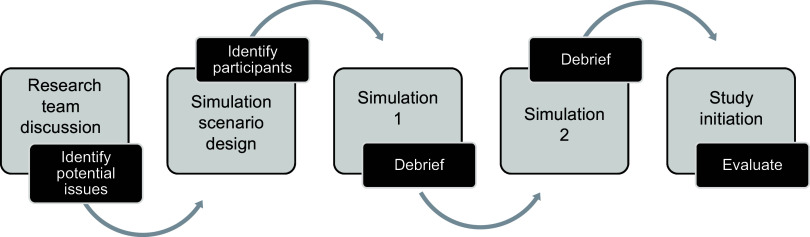
Overall depiction of the steps in developing, performing, and debriefing simulations prior to the initiation of complex clinical trials.

After a 10–15-minute orientation over videoconferencing to review the purpose of the session, the simulation commenced with in-person participation. Individuals representing each stakeholder group were drawn into the scenario at different times based on their specific roles. All participants and the simulation team took notes and jotted observations and questions that required clarification and discussion. Each simulation was conducted over approximately 60–90 minutes, followed by an additional 60 minutes for debriefing simulation events. The simulation team utilized a checklist comprised of anticipated scenario activities (Supplementary Material 2) to facilitate a semi-structured team debriefing, during which participants identified aspects of the simulation that occurred fairly smoothly, as well as obstacles that needed to be addressed. After each session, the core research team and simulation facilitator discussed findings, sought solutions to potential problems, and decided what materials had to be developed prior to initiation of the study. Audio recordings and/or detailed notes of all discussion points during the debriefing and post-debriefing discussions were compiled for reference and thematically categorized. An overview of the entire process is depicted in Figure 1.

## Results

Two separate simulations and debriefings were facilitated by the authors. Eleven individuals representing each of the stakeholder groups participated in the simulations in groups of 7–9 individuals; 3 of these individuals participated in the same roles in both sessions. All roles necessary to conduct the clinical study were represented for each simulation: representative neonatology fellow, bedside nurse, study investigator, 1–2 study coordinators, EEG technician, investigational pharmacist, and child neurologist. Discussions from the two team debriefings uncovered a total of 80 discrete questions, comments, and items that required clarification. The debriefing after the second scenario revealed 31 questions, with only 6 (19%) overlapping with those discussed after the first scenario. Questions and points for clarification were classified into 4 main thematic categories with subcategories (Table 1), which were utilized to improve the checklist used for process mapping.

**Table 1.** Study challenges identified during simulation-based rehearsal of study recruitment, enrollment, randomization, and initial study procedures

Theme	Subtheme	Description
Communication among stakeholders	Role clarity	Clarification of roles among the clinical and study teams prevents unnecessary duplication of work or the omission or incomplete performance of study procedures.
	Multidisciplinary coordination and communication	Clinical trials involving multiple stakeholders, particularly if there is a short window between the identification and randomization of potential subjects, must outline a clearly delineated plan for notification of involved services.
		The study coordinator identifies who should be involved in the screening process, the best methods to facilitate timely communication among stakeholders, and what communication is necessary to ensure accurate identification of potential participants.
Execution of study procedures	Population-specific considerations	Identification of potential subjects must be timely in studies with tight timelines.
	Workflows	Clinical workflows may require modification to safely and efficiently accommodate study-related procedures without complicated work-around processes that may cause errors.
	Adherence to timelines	Enrollment windows may preclude optimal subject accrual.
		Consent practices should be tailored for efficiency. These may include designating the person to request consent, approaching families for prenatal consent (for neonatal trials), developing materials that best inform potential subjects and/or families, and decreasing the initial approach-to-consent interval.
		Protocol violations may occur
	Time of day/day of week considerations	Staffing varies based on the time of day and day of week. Modification of study procedures and personnel may be necessary for weekends and nights.
	Maintenance of blinding	Stakeholders should verify the individuals who must remain blinded versus those who may need unblinded access for patient safety reasons.
		Simulation may uncover times when the clinical team may inadvertently become unblinded to a subject’s study arm assignment.
Resources	Personnel and equipment availability	Limitations related to the availability of personnel and equipment (e.g., EEG technicians, video EEG equipment, etc.) will affect the timely completion of study procedures.
	Tool development	Tools must be developed to improve understanding of study procedures so that tasks may be accomplished safely and efficiently (e.g., order sets in the patient’s electronic medical record).
	Electronic interfaces	The study team needs to familiarize themselves with any clinical and study-specific electronic systems to ensure the accurate and timely collection of data.
		Targeted simulations that focus on electronic systems may be conducted separately from larger team-based simulations.
Standards of care vs. study protocols	Prioritization of patient care	The study team (expert in study protocols) and clinical teams (expert in standards of care) need to work closely to identify potential conflicts between clinical guidelines and study protocol, particularly instances when clinical teams need to prioritize patient care for safety reasons.

EEG = electroencephalogram.

**Figure 2.**
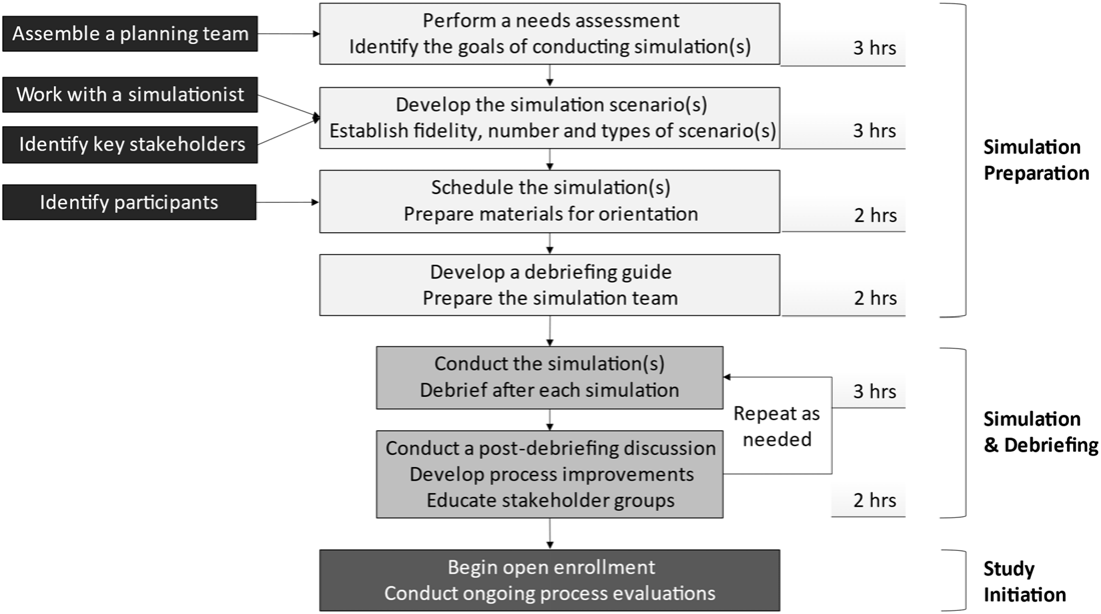
Detailed description of the steps involved in developing, performing, and evaluating team performance prior to the initiation of complex clinical trials. Time estimates are provided for each of the steps, though times will vary depending on the complexity of the trial, the number of roles, and the experience level of the clinical research team.

After conducting the simulations and analyzing the debriefings, the authors identified the potential benefits and considerations to best utilize simulation as a tool for successful clinical trial preparation (Fig. 2, Table 2, Supplementary Material 3).

**Table 2.** Identified benefits of utilizing simulation for clinical trials preparation

Benefits	Description
Stakeholder engagement	Key participating services become actively involved in solving potential barriers prior to study initiation
Relationship building	Prior to study initiation, involvement of all services allows relationship building to gain input on workflow, optimal routes of communication, identification of potential barriers.
Identification of challenges	A wide variety of challenges and strategies to overcome them can be identified prior to study initiation, such as factors that could cause enrollment failures or significantly delay study procedures; identification may decrease later protocol deviations and violations.
Rehearsal	Communication, screening, and enrollment practices, inter-team collaborations can be rehearsed in a less threatening and less high-stakes arena
Stakeholder training needs	Stakeholder training needs can be identified and planned for ahead of study implementation
Education	Individuals less familiar with the conduct of clinical trials, such as trainees, early-stage faculty, and new coordinators, can think through the steps meticulously and in a less threatening venue

## Discussion

In healthcare, simulation serves as a multifaceted tool to enhance individual and team-based training in classroom and clinical environments, as well as helps improve patient safety as a part of quality improvement initiatives. Simulation may also be applied as a valuable tool to help prepare study and clinical teams in the implementation of complex clinical trials.

The potential benefits of simulation are broad, ranging from stakeholder engagement and the promotion of effective team function to the identification of challenges that would negatively impact study procedures and/or subject safety (Table 2). In particular, immersing different stakeholders in team-based simulations, followed by team debriefings, can help uncover a greater number of potential issues than when conducting a “table-top” discussion that only involves discussion of the study protocol. After conducting two simulations, we identified 80 questions and challenges that required clarification and/or the development of strategies to improve subject enrollment and study implementation that were not immediately apparent from reading the Manual of Procedures.

The potential benefit of helping the study team anticipate issues is similar to that noted when employing simulation in quality improvement initiatives to identify latent safety threats that could lead to poor team function, near misses in patient care, or adverse patient outcomes [3,14,15]. In the clinical environment, simulation has helped to identify minor-to-major issues before embarking on a high-acuity, low occurrence clinical situation, initiating a new workflow, or moving clinical care into a new healthcare environment [1,6,7,9,11].

While all clinical trials may benefit from simulation, conducting simulations requires advanced planning and can utilize significant resources if the simulations are designed to be realistic in nature. When considering the investment of personnel, time, and other resources, simulation may be the most high yield with a return on investment for complex clinical trials that involve the coordination of personnel from multiple services and those that are very time sensitive. Outpatient studies that have the luxury of more time to obtain informed consent and more time between visits may not need this degree of preparation. However, inpatient studies in which potential subjects must be identified at any time of day or night, approached for consent within a limited window of time, and require the cooperation of individuals responsible for different aspects of the trial, would benefit from this type of planning and preparation. Going through the steps, rather than merely reading them from a Manual of Procedures, will identify potential barriers, complexities, and contingencies that are not readily apparent. Many questions may be resolved by querying the principal investigator or study sponsor. However, more challenging issues may require creative site-specific solutions, such as the development of new communication strategies, workflows, and/or educational materials. While additional resources would be required, testing identified solutions in a follow-up simulation is important to check their viability.

Advanced planning with different stakeholders, as well as considerations on how the simulations should be designed and conducted (Fig. 2, Supplementary Material 3), is necessary for the simulations to successfully achieve their intended aims. For multicenter studies, we suggest that the primary site should develop the scenario(s) for one or more simulation sessions. The overall principal investigator and their study team should pilot the simulation and not only critique their team’s performance but also identify potential hurdles and solutions. These scenarios, with modifications as necessary, can then be incorporated into site orientation and suggested for use at the individual sites with local personnel. Depending on institutional experience with simulation, study personnel, or “simulationists,” who are not involved with the study but have expertise conducting simulations in healthcare, can take the lead in developing and conducting these sessions.

The number of different scenarios and the number of times the scenarios are performed will vary based on many factors, including the time and resources needed to do the simulations, the participating team’s level of experience working together on clinical trials, the study’s complexity, and the range of subjects who may be eligible to participate. In general, a greater number of sessions with different scenarios would help study teams better capture the breadth of potential issues, especially when preparing for more complex clinical trials. For us, two simulations uncovered many questions and potential issues within 4 main thematic categories that required discussion. While participants in the second simulation were able to ask questions and identify issues that were distinct from those identified in the first simulation, they did not uncover new themes or subthemes (Table 1). For the clinical trial for which we were conducting simulations, two simulations may have been sufficient to help us confidently anticipate major issues. For other types of clinical trials, additional simulations may be necessary.

### Limitations

Before we were able to open local enrollment in the study for which we designed the simulations, the study sponsor decided to close the study, which prevented us from measuring the impact of the simulations. While the study sponsor did not disclose the reason for termination, we could speculate that the study was closed due to unacceptably slow enrollment since our site was contacted after the study was underway. Though we were unable to assess the value of the simulations, we speculate that we may have been able to achieve a higher rate of enrollment by decreasing the number of families not approached in a timely manner. There were several different ways we considered evaluating the effectiveness of simulations. In a clinical trial in which sites are randomized to utilize simulation versus their usual approach to study start-up, it may be possible to quantify the rates of protocol deviations and violations. The number of protocol deviations and violations would be expected to decrease with the use of simulations. As noted above, enrollment rates may also be improved, particularly in trials that require rapid identification, consent, and randomization of subjects. Less easily quantifiable, but also important, are stakeholder ratings of satisfaction or frustration during the course of the study. In our situation, sites were not randomized, but we may have been able to compare our performance on these metrics with that of the other participating sites. Clearly, there would have been other factors influencing successful study execution, but it may have served as a proof of concept and led to a more formal assessment of simulation for clinical trial start-up.

## Supporting information

Dadiz et al. supplementary material Dadiz et al. supplementary material

## Supplementary material

For supplementary material accompanying this paper visit https://doi.org/10.1017/S2059866124005594. click here to view supplementary material
