# Supplementary material for: Simulation as a potential tool for successful clinical trial initiation
Source: J Clin Transl Sci. 2024 May 31;8(1):e109. doi: 10.1017/cts.2024.559 (PMC11626590; doi:10.1017/cts.2024.559)
Supplement: Dadiz et al. supplementary material [file S2059866124005594sup001.docx]

**Supplementary Material**

1. **Simulation scenarios**

INSTRUCTIONS TO PARTICIPANTS

Your involvement in this simulation and debriefing will help the study team identify questions and potential challenges related to how the study should be conducted to optimize study efficiency and success while preserving subject safety. This session will be the first of two planned simulations before the study officially begins.

Please review highlighted areas of the study protocol (provided separately). Also find the agenda (see below), which provides the aims, participant roles, and an outline of anticipated study tasks to be completed during the simulation. The general agenda will be as follows:

15 minutes - Orientation

60 minutes - Simulation in the neonatal intensive care unit

60 minutes - Debriefing

During the simulation, please jot lots of notes and questions that you may have. We will discuss your observations immediately after the simulation during the debriefing.

PURPOSE OF SIMULATION

1. To identify potential challenges and strategies to address workflow issues of conducting the phenobarbital study.
2. To estimate the amount of time needed to perform the different tasks during the Screening Period and part of the Double-Blind Treatment Period of the study.
3. To identify ways of improving the efficiency of performing study-related tasks.

SESSION PARTICIPANTS

1. Simulation team: simulation facilitator and core research team
2. Observer(s)
3. Simulation participants, listed in the order of first appearance in the simulation:
   1. Clinical team: fellow, advanced practice provider, and nurse
   2. Research team: site investigator and study coordinator
   3. Parent (portrayed by actor)
   4. Encephalography (EEG) technician
   5. EEG specialist or neurologist
   6. Investigational Drug Service (IDS) pharmacist

SCENARIO #1: Hypoxic Ischemic Encephalopathy

Brief Infant Synopsis

Name: Hill, Girl

Gestational age: 40 weeks

Birth weight: 3000 grams

This is a full-term female infant with hypoxic ischemic encephalopathy who was born 1 hour ago via vaginal delivery.

Maternal History

Name: Hill, Brittany

Age: 35 years

Obstetric history: Gravida 1, Parida 0

Brittany has type II diabetes, for which she takes insulin. Her prenatal labs are normal.

Delivery History

Apgar scores: 1, 2, 4

Arterial cord gas: pH 6.95, pCO_2_ 75, base deficit -16

The delivery was complicated by a three-minute shoulder dystocia, which required obstetric maneuvers to deliver the infant. Upon delivery, the infant was limp, cyanotic, and had no respiratory effort. The heart rate was 55 beats per minute (bpm). The neonatal intensive care unit (NICU) team provided positive-pressure ventilation via a self-inflating bag. The heart rate improved gradually to >100 bpm by 1 minute of life (MOL). However, the infant continued to have minimal respiratory effort, poor tone, and no spontaneous grimace. She was intubated at 2 MOL.

Current Status

The infant is on mechanical ventilation, requiring moderate settings and oxygen delivery. The arterial blood gas 30 minutes after birth showed: pH 7.02, pCO_2_ 50, base deficit -14. The chest radiograph shows no acute pulmonary disease with appropriate cardiothymic silhouette. She is receiving intravenous fluids via central umbilical catheters.

SCENARIO START AND END POINTS

The scenario commences with the clinical team examining the infant and performing a risk assessment for potential seizures. The simulation will end after the study drug loading dose is administered and the pharmacokinetic (PK) labs are ordered.

SCENARIO #2: Acute onset of seizures/meningitis (off-hours)

Brief Infant Synopsis

Name: James, Boy Gestational age: 40 weeks Birth weight: 3000 grams

This is a full-term male infant who was admitted to the NICU for a sepsis evaluation due to an elevated Kaiser sepsis risk score.

Maternal History

Name: James, Maria Age: 35 years Obstetric history: Gravida 1, Parida 0

The mother had prolonged rupture of membranes for 24 hours with a fever during labor. Her prenatal labs were significant for unknown Group B streptococcus status.

Delivery History

Apgar scores: 7, 8 Cord gases: not done

The infant was born via vaginal delivery at 12:13 pm. Upon delivery, the infant was vigorous with HR >100 bpm. The delivery room team warmed, dried, stimulated and bulb suctioned the infant. The infant’s color improved. He was admitted to the NICU for a sepsis evaluation and close observation due to an elevated Kaiser sepsis risk score.

Initial NICU Course

A peripheral iv was placed after admission, with 10% dextrose solution at a rate of 60 ml/kg/day. After obtaining a complete blood count and a blood culture, empiric ampicillin and gentamicin were started. The infant initially did not require respiratory support. However, he started having apneic episodes at 15 hours of age and was placed on high flow nasal cannula at 4 liters per minute, 21% O2. A lumbar puncture was performed, and cerebrospinal fluid was sent for cell count, chemistries, and culture. The infant had an episode of seizure-like activity. It consisted of eye deviation to the right, lip smacking, and increased tone of the upper and lower extremities. Subsequently, the blood culture grew Group B streptococcus. Ampicillin was changed to meningitic dosing.

SCENARIO START AND END POINTS

The scenario commences with the clinical team examining the infant, recognizing the occurrence of seizures (apneic events and/or eye deviation, lip smacking and increased tone) and contacting the research study team. The simulation will end after the study drug loading dose is administered and the study laboratories are ordered.

1. **Checklist of anticipated activities**

| **Time** | **Study Task** | **Observer Notes** |
| --- | --- | --- |
| **If the patient has an increased risk for developing a seizure** | | |
|  | 1. Clinical team conducts a physical exam and risk assessment for potential seizures. |  |
|  | 1. Clinical team notifies study coordinator of potential study subject. |  |
|  | 1. Clinical team calls the EEG technician and specialist to obtain EEG monitoring and give a heads-up for potential study enrollment. 2. EEG technician places EEG leads and starts monitoring. |  |
|  | 1. Study coordinator reviews subject eligibility, then closes the communication loop with NICU team. |  |
| **If the patient meets study eligibility** | | |
|  | 1. If the infant meets study eligibility, the study coordinator calls IDS. |  |
|  | 1. Research team approaches the family about the study and obtains consent. |  |
|  | 1. Clinical team conducts study physical exam. 2. Clinical team orders baseline study labs. |  |
|  | 1. Study coordinator enters subject into the IWRS. |  |
|  | 1. IDS prepares the study drug for both study arms. 2. IDS places the study drug into the medication dispensing system. 3. IDS informs the study coordinator after completion of study drug preparation. |  |
| **Fast forward... If the infant has clinical seizure-like activity** | | |
|  | 1. Clinical team notifies the study coordinator. 2. Study coordinator contacts the EEG specialist or neurologist. 3. EEG specialist or neurologist reviews the EEG and confirms whether there is an electrographic seizure. 4. EEG specialist or neurologist informs the study coordinator. |  |
| **If the infant has confirmed electrographic seizure** | | |
|  | 1. Study coordinator completes the randomization process in the IWRS. |  |
|  | 1. Study coordinator records the randomization and study drug syringe code. 2. Study coordinator communicates the code to the NICU team as a group text. |  |
|  | 1. Clinical team orders the study drug. |  |
|  | 1. Per randomization, the clinical team obtains the study drug loading dose from the medication dispensing system. |  |
|  | 1. Clinical team administers the study drug loading dose to the subject. |  |
|  | 1. Clinical team orders the PK labs. 2. Clinical team collects and sends PK labs after the study drug loading dose per PK group assignment. |  |

Abbreviations: EEG, electroencephalogram; IWRS, interactive web response system; NICU, neonatal intensive care unit; PK, pharmacokinetic

1. **Detailed framework and considerations of utilizing simulation for clinical trials preparation**

| **Preparation Step** | **Considerations for Simulation Planning** | **Approximate Time to Complete (Primary Person Responsible)** |
| --- | --- | --- |
| Assemble a planning team to develop and conduct the simulations. | The principal investigator and study coordinator identify the simulation specialist(s) to help achieve the goals of simulation. Based on the type of study, the team may identify other essential individuals. | 1 hour (PI and coordinator) |
| Perform a needs assessment | The planning team reviews the study protocol in detail to ensure understanding of study procedures and to anticipate areas of potential challenges that would require viable solutions. Outlining and mapping the steps that stakeholders would undergo during the study helps organize planning. | 2-4 hours per core team member (all), depending on complexity of trial, length of the protocol, and experience of team member |
| Identify the goals of conducting the simulation | The identified challenges guide the development of learning objectives. | 1 hour (core team) |
| Develop the simulation scenarios | The anticipated challenges serve as the foundation for developing the simulation scenarios. Based on the number and types of challenges, the planning team would need to identify:   - The specific complexities that will be covered in each scenario; - The number of scenarios to be developed; - The length of each scenario; - The conditions that may differ in the various scenarios.   Examples of conditions that may be modified to test different types of challenges include:   - The patient’s clinical presentation and/or diagnosis; - The time of day when the patient presents as a potential subject.   If a study protocol is particularly complex with different study procedures and/or involvement of many different types of stakeholders, the planning team may consider developing shorter simulations that focus on different aspects of the study rather than run a single large simulation that encompasses all parts of the study. | 2-4 hours (PI and simulationist), depending on complexity and experience of core team members |
| Establish the level of fidelity that should be achieved during the simulations | The planning team should decide how realistic the simulation needs to be (“fidelity”) to identify challenges and achieve the goals of the simulation. Considerations include asking the following questions:   - Should the simulation take place in the clinical environment, or would a conference room suffice? - Do the events of the simulation need to occur in real time? - What equipment and supplies are needed? - Is a virtual electronic medical record for the patient necessary? - Is an actor to portray the patient or family member needed? | 1-2 hours (PI and simulationist) |
| Identify the key stakeholders who should participate in the simulations | Stakeholders should comprise all the different professional groups who will have an active, frontline role in performing the various study-related tasks. | 1 hour (PI) |
| Schedule the simulations | As part of scheduling, the planning team needs to coordinate the schedules of many individuals representing the different stakeholders whose roles are integral to the successful implementation of the study. The planning team will need to estimate the number of simulations needed to achieve their goals and objectives. While one simulation may not be enough to test the different conditions that stakeholders may encounter in actual practice, conducting the simulations until no new issues emerge may be an excessive use of resources and time. | 2 hours (coordinator) |
| Prepare materials for stakeholder orientation and tools that may be utilized during the simulations | The planning team should communicate the goals and learning objectives to stakeholders before they participate in the simulations so that they are prepared to help identify challenges and potential solutions. Materials necessary for the simulation should be distributed in advance. | 1-2 hours, depending on number of stakeholders involved in the simulation (simulationist) |
| Develop a debriefing guide | The debriefing guide consists of a series of questions to help facilitate group discussion after stakeholders participate in the simulation. Questions should reflect the learning objectives that the planning team would like to achieve. | 2 hours (simulationist) |
| Prepare the simulation team | The planning team should prepare and designate roles for the individuals who will participate in the simulations. These individuals may, but do not need to be, the same individuals as on the planning team. Roles would include the following:   - Lead facilitator: orients participants, orchestrates the simulation, and redirects participants during the simulation to keep the simulation running efficiently and smoothly - Debriefer(s): facilitates a group discussion on their experiences after stakeholders participate in the simulation - Observer(s): observes the simulation and debriefing, takes notes on observations and discussion points | 1 hour (core team) |
| Conduct the simulation | The simulation team orients the participating stakeholders, then the simulation scenario commences until the designated end point is reached. | Orientation 15 minutes  Simulation: 60 minutes |
| Conduct the debriefing to identify challenges | The debriefer(s) facilitates a semi-structured group discussion after each simulation, utilizing the debriefing guide. Participants are asked to identify challenges they encountered, other salient points, and potential strategies that would help the research team implement the clinical trial successfully. Discussing events chronologically helps organize participants’ thoughts so that key events are not missed. There are different models of debriefing that debriefer(s) may utilize to structure debriefings [1]. | Debriefing: 60 minutes |
| Conduct a post-debriefing discussion among the simulation team | After the debriefing, the simulation team and core research team discuss debriefing points, develop plans for the next simulation, formulate initial strategies to resolve identified issues. | 2 – 3 hours (core team, simulation team) |

1. Abulebda K, Auerbach M, Limaiem F. Debriefing techniques utilized in medical simulation. *StatPearls*. Treasure Island, FL: StatPearls Publishing, 2023 Jan.
